# *In vivo* antimicrobial activity of marbofloxacin against *Pasteurella multocida* in a tissue cage model in calves

**DOI:** 10.3389/fmicb.2015.00759

**Published:** 2015-07-24

**Authors:** Changfu Cao, Ying Qu, Meizhen Sun, Zhenzhen Qiu, Xianhui Huang, Binbin Huai, Yan Lu, Zhenling Zeng

**Affiliations:** ^1^National Reference Laboratory of Veterinary Drug Residues, College of Veterinary Medicine, South China Agricultural UniversityGuangzhou, China; ^2^National Laboratory of Safety Evaluation (Environmental Assessment) of Veterinary Drugs, College of Veterinary Medicine, South China Agricultural UniversityGuangzhou, China

**Keywords:** marbofloxacin, *Pasteurella multocida*, tissue cage model, *in vivo* antimicrobial activity, calves

## Abstract

Marbofloxacin is a fluoroquinolone specially developed for use in veterinary medicine with broad-spectrum antibacterial activity. The objective of our study was to re-evaluate *in vivo* antimicrobial activity of marbofloxacin against *Pasteurella multocida* using subcutaneously implanted tissue cages in calves. Calves were infected by direct injection into tissue cages with *P. multocida*(type B, serotype 2), then intramuscularly received a range of marbofloxacin doses 24 h after inoculation. The ratio of 24 h area under the concentration-time curve divided by the minimum inhibitory concentration or the mutant prevention concentration (AUC_24__h_/MIC or AUC_24__h_/MPC) was the pharmacokinetic-pharmacodynamic (PK/PD) index that best described the effectiveness of marbofloxacin against *P. multocida* (*R*^2^ = 0.8514) by non-linear regression analysis. Marbofloxacin exhibited a good antimicrobial activity *in vivo*. The levels of AUC_24__h_/MIC and AUC_24__h_/MPC that produced 50% (1.5log_10_ CFU/mL reduction) and 90% (3log_10_ CFU/mL reduction) of maximum response were 18.60 and 50.65 h, 4.67 and 12.89 h by using sigmoid *E_max_* model WINNONLIN software, respectively. The *in vivo* PK/PD integrated methods by tissue cage model display the advantage of the evaluation of antimicrobial activity and the optimization of the dosage regimen for antibiotics in the presence of the host defenses, especially in target animal of veterinary interest.

## Introduction

To optimize antimicrobial therapy that maximizes efficacy and minimizes selection of resistance, the pharmacokinetics (PKs) and pharmacodynamics (PDs) characteristics need to be integrated. Recently, a variety of *in vitro* and animal models that fractionated dosage regimens showed that patterns of antimicrobial activity can be divided into two major patterns: time-dependent killing and concentration-dependent killing (Jacobs, 2001; Toutain et al., 2002; Drusano, 2004). Time-dependent killing is characteristic of β-lactams, macrolides, and clindamycin, and seeks to optimize the duration of exposure of a pathogen to an antimicrobial. Their major PK/PD parameter correlating with effectiveness is the percentage of the dosing interval that the antibiotic concentration exceeds the minimum inhibitory concentration (%T > MIC). The concentration-dependent killing, such as quinolones and aminoglycosides, seeks to maximize antimicrobial concentration, and their major PK/PD parameter correlating with effectiveness is the ratio of 24 h area under the concentration-time curve or the peak antibiotic concentration divided by major susceptible cell (MIC; AUC_24__h_/MIC or C_max_/MIC).

Many *in vitro* and *in vivo* studies have demonstrated that fluoroquinolone-resistant subpopulations are selectively enriched in the mutant selection window (MSW), the concentration range between MIC and mutant prevention concentration (MPC; Cui et al., 2006; Olofsson et al., 2006; Drlica and Zhao, 2007). The lower boundary of the MSW is approximately the minimal concentration that inhibits the growth of the MICs. And the upper boundary is the MPC, defined as the lowest antibiotic concentration that prevents growth of the least-susceptible single-step mutant subpopulation among a large (10^10^ cfu) bacterial population (Drlica and Zhao, 2007). Consequently, recent studies have proposed that MPC-based PK/PD indices such as AUC_24__h_/MPC or C_max_/MPC are more appropriate than MIC-based indices for predicting mutant-restricting fluoroquinolone doses (Olofsson et al., 2006; Liang et al., 2011), because MPC is a direct measure of mutant subpopulation susceptibility while MIC is not, and correlates poorly with MPC (Liang et al., 2011). Thus, MPC-based PK/PD indices can be very useful PD parameters for optimization of antimicrobial therapy.

Recently, a variety of models have been developed to study the relationship between the PKs and PDs. However, each model has its own limitations, e.g., for *ex vivo* PK/PD integrated model, there is continuous exposure to static concentration of agent for a defined time(24 h), which does not mimic the concentration of antibacterial decline in animals for body clearance and drug metabolism(Xiao et al., 2015). *In vitro* models do not account for effects of interaction between the host and the infecting organism (MacGowan and Bowker, 2002). The infection models in small rodents (mice, rats or rabbits) are hampered by differences in PK parameters between small rodents and larger animals in that they may exist in complicate extrapolation of results to the clinical situation (Zak and O’Reilly, 1991). Nevertheless, tissue cages implanted subcutaneously attract some attention for PKs and PDs studies, especially in a target animal of veterinary interest (Clarke, 1989). Because subcutaneously implanted tissue cages allow for repeated sampling, this model has been used extensively to study the distribution of antimicrobials(Clarke, 1989) and *ex vivo* antibacterial effectiveness in animal species of direct veterinary interest (Aliabadi and Lees, 2002; Potter et al., 2013; Shan et al., 2014). In addition, tissue cage model has been used to contain an infection to allow for *in vivo* studies of antibacterial effectiveness (Greko et al., 2003a,b) and MSW studies in presence of the host defenses (Zhao and Drlica, 2001; Cui et al., 2006; Ni et al., 2014).

Marbofloxacin, a synthetic third-generation fluoroquinlone, is specifically developed for individual veterinary treatment with a broad spectrum of activity against most gram negative bacteria, gram positive bacteria, *mycoplasmas*, and some intracellular pathogens such as *Chlamydia* and *Brucella species* (Spreng et al., 1995). For antimicrobial activity properties of marbofloxacin, studies reported the values of AUC_24h_/MIC required three levels of antibacterial activity, e.g., bacteriostatic, bactericidal and virtual eradication by *ex vivo* PK/PD integrated model in different biological fluids, respectively, in species of veterinary interest(Aliabadi and Lees, 2002; Sidhu et al., 2011; Shan et al., 2014). However, to our knowledge, *in vivo* antibacterial activity of marbofloxacin against *Pasteurella multocida* has not been reported in target animal-calves.

The main goal of our study was to re-evaluate *in vivo* antimicrobial activity of marbofloxacin against *P. multocida* based on MIC and MPC in a tissue-cage model in calves.

## Materials and Methods

### Antimicrobial Agent

Marbofloxacin was used as 10% injectable aqueous solution obtained from Veterinary Pharmaceutical Corporation (Yuanzhen Co., Ltd, Hebei, China). Marbofloxacin standard was purchased from Dr.Ehenstorfer GmbH Company (Germany) and ofloxacin was acquired from the National Institute for Food and Drug Control (Beijing, China).

### Bacterial Strain

Strain CVCC1669 (type B, serotype 2) of *P. multocida* was obtained from the China Veterinary Culture Collection Centre (Beijing, China). The original bacterial culture had been isolated from a calve that died of hemorrhagic septicemia in the United States. The organism was grown, sub-cultured and quantified in Mueller–Hinton Π Broth (Becton Dickinson, Sparks, MD, USA) and Tryptic Soy Agar (Guangdong Huaikai Microbial Sci. & Tech. Co., Ltd, Guangzhou, China) supplemented with defibrinated sheep blood (BTSA) at a 5% level.

### Tissue-Cage Infection Model

**FIGURE 1:**
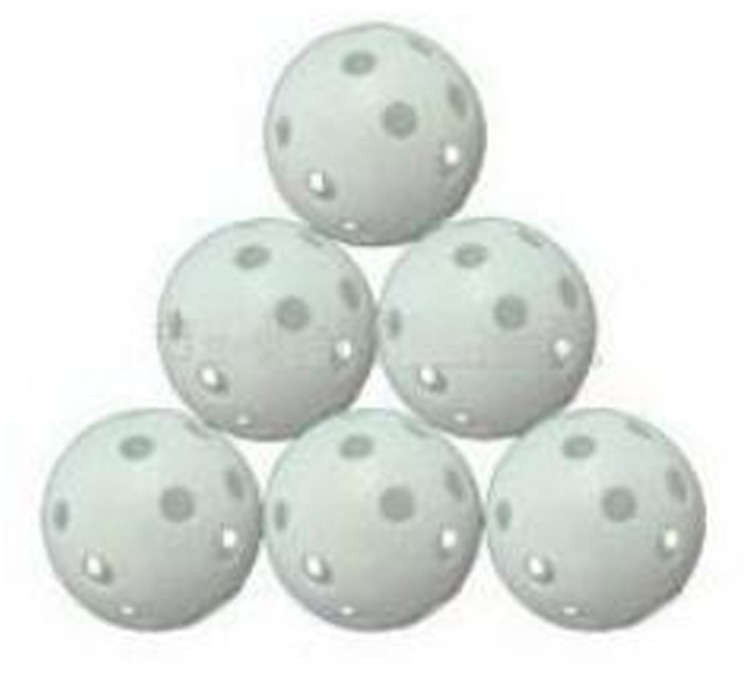
**The tissue cages used in *in vivo* antimicrobial activity of marbofloxacin against *Pasteurella multocida* in calves**.

Two sterile golf practice wiﬄe balls (43 mm in diameter, with a volume of 34 ml, as shown in **Figure 1**) were implanted subcutaneously in calves, after sedation and local anesthesia. The procedures were as described elsewhere (Aliabadi and Lees, 2002; Greko et al., 2003a). After surgery, all calves were treated with intramuscular penicillin (*160000 IU/kg*) twice a day for 3–5 days to prevent infection. By 4 weeks after implantation, each tissue-cage had become sealed with a thin layer of connective tissue and filled with clear, yellowish tissue cage fluid (TCF). Sampling of TCF was performed by percutaneous puncture. Sterility of TCF was assessed by aerobic and anaerobic culture of samples taken from each cage immediately before the experiments. Approximately 5 × 10^6^ CFU of exponentially growing *P. multocida* culture was suspended in 100 μl of sterile isotonic saline, and then injected into the pre-implanted each wiﬄe ball.

Tissue cage fluid was sampled after inoculation(-24 h) corresponding to the time of the injection of drug (0 h) and 3, 6, 9, and 24 h after administration. Each sample (50 μl) was subjected to 10-fold serial dilutions using 0.9% NaCl. Twenty five micro liter of each dilution was plated onto quadrants of BTSA agar. The plates were then incubated at 37°C overnight and viability counts were examined. The limit of detection (LOD) was 400 CFU/mL.

### *In Vitro* Susceptibility Studies

The MIC value was determined in TCF by a broth microdilution assay based on Clinical and Laboratory Standards Institute (CLSI) reference methods using the following two overlapping sets of twofold serial dilutions to improve accuracy: 0.0125–0.4 μg/mL and 0.0182–0.3 μg/mL.

The MPC value was determined as described elsewhere (Campion et al., 2004; Ferran et al., 2011). Single bacterial colony from overnight growth on Tryptic Soy Agar supplemented with defibrinated sheep blood (BTSA) was grown for 5 h at 37°C in Mueller–Hinton Π Broth, concentrated by centrifugation, and re-suspended in Mueller–Hinton Π to a final concentrations of ∼3 × 10^10^ CFU/mL. Samples (100 μl) were then plated onto Mueller–Hinton Agar (MHA; supplemented with defibrinated sheep blood) containing various concentrations of marbofloxacin obtained by successive twofold dilutions, inoculated plates were incubated for 24 h at 37°C and then examined for regrowth. All plates were re-incubated for an additional 24 h and rescreened. MPC_pr_ was recorded as the lowest antibiotic concentration that allowed no growth. This measurement was followed by a second measurement that utilized linear drug concentration increments that did not exceed 20% per sequential increase. These determinations were performed in triplicates.

### Determination of Protein Binding *In Vitro*

Protein binding studies were performed in triplicate using Amicon Centrifree Micropartition devices (Millipore, Bedford, MA, USA) with 10000 Nominal Molecular Weight Limit according to the manufacturer’s package insert. Pooled uninfected TCF was spiked with 0.05, 0.1, 0.5, 1, and 2 μg/mL marbofloxacin. The concentrations were selected based on TCF concentration profiles of the doses that were utilized in the PD studies. Five hundred micro liter was added into a ultrafiltration device and then contrifugated at *4000 g* for *45 min* at 25°C to generate an ultrafiltrate of ∼250 μl. Protein binding of marbofloxacin was also determined in bovine serum spiked with the same concentration as TCF, but in this case, protein-bound drug was removed by ultracentrifugation at 12000 *g* for 45 min at 25°C. The ultrafiltrate was frozen at -20°C until assayed.

The percentage of protein bound fraction (%PB) was calculated according to the following equation: %PB = (initial concentration-ultrafiltrate concentration)/initial concentration × 100%. The drug free fraction (*fu*) = 1-%PB.

### Animal

Eighteen female calves of Luxi Yellow Cattle (∼8 months with a mean weight ± SD of 145 ± 24 kg) were used in the study. All animal studies were approved by the animal research committees of the South China Agriculture University. The animals were maintained in accordance with the American Association for Accreditation of Laboratory Animal Care Criteria.

### Pharmacokinetic Measurements

Eighteen calves were divided randomly into nine administration groups (two calves, four tissue-cages/group), which received a series of dosages such as 0, 0.3, 0.5, 0.8, 1.0, 1.3, 1.6, 2.0, and 2.8 mg/kg of body weight once daily intramuscularly after inoculation (-24 h). This series of dosages achieved values of AUC_24__h_/MIC for bacteriostatic action, bactericidal action, bacterial elimination by assuming that the PK parameter of marbofloxacin is proportional to the dose (Aliabadi and Lees, 2002; Shan et al., 2014) and were determined by preliminary experiments. TCF (0.5 ml) was removed from the wiﬄe balls by percutaneous puncture at 0, 1, 3, 6, 9, 12, 24, 30, 36, and 48 h after marbofloxacin administration. Fluid samples were clarified by centrifugation 6000 *rpm* for 10 min and stored at -20°C. The total concentration of marbofloxacin was determined by high-performance liquid chromatography (HPLC; Aliabadi and Lees, 2002; Shan et al., 2014). The lower LOD was 0.005 μg/ml. Marbofloxacin quantitation from TCF and ultrafiltrate were linear within a range of 0.01–5 μg/ml. The recovery of marbofloxacin was >80%, and relative standard deviations for both interday and intraday were <10% in TCF.

Pharmacokinetic parameters obtained from the marbofloxacin concentration data from each cage, including area under the concentration-time curve over 24 h (AUC_24__h_), and the peak drug concentration in TCF (C_max_), were calculated by a non-compartment model using WinNonlin 6.2 software (Pharsight Corporation, Mountain View, CA, USA) and Microsoft Excel was used to estimate %T > MIC values.

### Pharmacokinetic-Pharmacodynamic (PK/PD) Analysis

By using the individual PK parameter data obtained from each cage, the relationship between drug exposure and bacterial response was expressed according to the following parameters: the ratio of peak drug concentration divided by MIC (C_max_/MIC) or MPC (C_max_/MPC), the ratio of 24 h area under the curve of the TCF concentration over MIC (AUC_24__h_/MIC) or MPC (AUC_24__h_/MPC) and the percentage of the dosing interval that TCF drug concentrations were above the MIC or MPC (%T > MIC or %T > MPC). The correlation between antimicrobial effectiveness and each of the PK/PD parameters were determined by non-linear regression analysis (Origin software, Version 9.2). The *in vivo* PK/PD relationship of marbofloxacin was described using sigmoid *E_max_* model WinNonLin 6.2 software. The equation was described as the following: *E = E_max_-(E_max_-E_0_) × C_e_^N^/(EC_50_^N^+ C_e_^N^)*, where *E* is the antibacterial effect, measured as the change in the bacterial counts 24 h after administration from the injection of marbofloxacin(ΔLog_10_ CFU/mL), *E_max_* is the change in the infected tissue-cage between 0 and 24 h after the treatment of saline. *E_0_* is the maximum change after i.m administration which represents the maximum antibacterial effect. *C_e_* is the AUC_24__h_/MIC or AUC_24h_/MPC parameter. *EC_50_* is the *C_e_* value required to achieve 50% of the *E_max_*. *N* is the Hill coefficient that describes the steepness of the AUC_24__h_/MIC or AUC_24__h_/MPC-effectiveness curve.

## Results

### *In Vitro* Susceptibility Testing

The MIC and MPC of marbofloxacin against *P. multocida* was 0.075 μg/mL, 0.3 μg/mL, respectively, and the ratio of MPC/MIC was 4. The MICs for the post-treatment isolates recovered from tissue cages did not change 24 h after exposure to marbofloxacin therapy.

### Protein Binding *In Vitro*

The protein binding of marbofloxacin in TCF and serum was 40 ± 7%, 52 ± 4%, respectively, when marbofloxacin concentrations ranged from 0.05 to 2 μg/mL (**Table 1**). Obviously, the characteristic of the protein binding was not concentration dependent. The protein binding can account for the differences in MIC determined in Mueller–Hinton broth (MHB) and in biological fluids such as serum, interstitial fluid. Generally MIC_MHB_ = *fu*MIC_biological_ _fluids_, where *fu* is the fraction of unbound marbofloxacin in the biological fluids.

**Table 1:** The protein binding of marbofloxacin in TCF and serum *in vitro.*

Spiked concentration (μg/ml)	*In vitro* Protein binding( 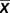 ± SD)	
	TCF^∗^	Serum
0.05	0.40 ± 0.08	0.52 ± 0.05
0.1	0.37 ± 0.09	0.53 ± 0.04
0.5	0.41 ± 0.07	0.49 ± 0.04
1	0.40 ± 0.06	0.51 ± 0.05
2	0.43 ± 0.08	0.55 ± 0.03
**Total**	0.40 ± 0.07	0.52 ± 0.04

*^∗^TCF, tissue cage fluid.*

### Tissue-Cage Infection Model

All tissue cages were sterile before the start of the experiments, and no severe illness or distress occurred during the experiment period. However, an increase in the rectal temperature (fluctuation range from 38 to 40.8°C) was noted in all calves during the experiments and The main inflammation reaction is swelling through macroscopic observation, and touching the skin around the infected tissue cages, the performance of the pain and febrile was observed 12 h after inoculation. A total of four tissue cages in three calves were excluded because of hemorrhaging.

The geometric mean bacterial counts in TCF from the cages at 24 h after inoculation increased to 8.12 ± 0.31 log CFU/mL from ∼5 × 10^6^ CFU/mL. The geometric mean for all non-treated cages at the end of the experience (48 h) was 8.83 ± 0.17 log CFU/mL.

### Marbofloxacin *In Vivo* Antibacterial Activity

**FIGURE 2:**
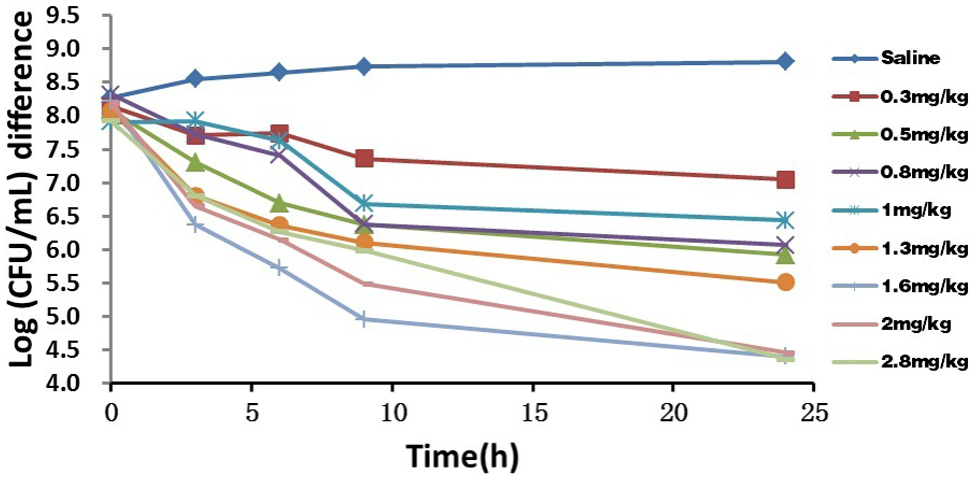
***In vivo* antimicrobial activity of marbofloxacin against *Pasteurella multocida* in tissue-cage model after i.m administration at various doses**. Bacterial counts in tissue-cage fluid was monitored at 0, 3, 6, 9, and 24 h after the treatment of marbofloxacin. Data for SE of the mean values were excluded for clarity.

The *in vivo* antibacterial activity of marbofloxacin against *P. multocida* (CVCC 1669) had a relatively good effectiveness 9 h after i.m administration, but subsequently only a slight reduction or inhibitory effect as shown in **Figure 2**. No re-growth occurred within 24 h exposure after treatment. The doses required to decline by ∼4 log_10_ CFU/mL ranged from 1.6 to 2.8 mg/kg, whereas the mean bacterial counts in TCF of untreated controls increased by ∼0.7 log_10_CFU/mL. Although bacterial count presented a downward trend after administration at relatively lower doses, the local inflammatory reactions (e.g., febrile, swelling and festering) become more serious. Considerable variation in the bacterial count among tissue cages after administration at various doses was observed. This is due to infection that influences vascular supply differently and other factors that determine the elimination rate from the tissue cages. This was consistent with the observation for other antimicrobials (Greko et al., 2003a,b).

### Correlation of PK/PD Parameters with Effectiveness

**FIGURE 3:**
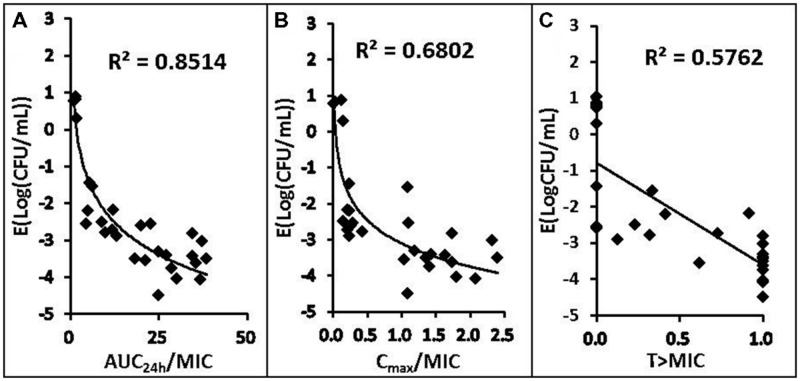
**Relationships between PK/PD parameters based MIC for *Pasteurella multocida* (CVCC1669) and Δlog_10_ CFU/mL after 24 h of therapy in the infected tissue-cage.** The lines represent the best model fits of the data. *R*^2^ is the correlation coefficient.

**FIGURE 4:**
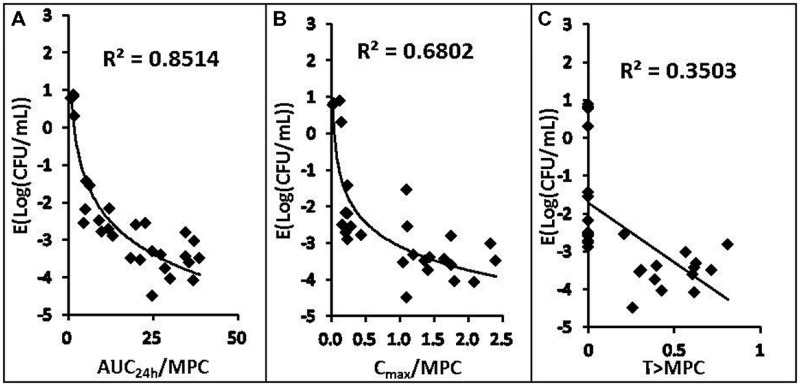
**Relationships between PK/PD parameters based MPC for *Pasteurella multocida* (CVCC1669) and △ log_10_CFU/mL after 24 h of therapy in the infected tissue-cage.** The lines represent the best model fits of the data. R^2^ is the correlation coefficient.

The relationships between the antibacterial effectiveness (Δlog_10_ CFU/mL: a reduction of the count of *P. multocida* (CVCC 1669) after marbofloxacin treatment compared to before treatment) and each of the PK/PD parameters are shown in **Figures 3** and **4**. The parameter AUC_24__h_/MIC and AUC_24__h_/MPC correlated significantly with effectiveness (*R*^2^ = 85.14%), whereas regression of the other PK/PD parameters versus the antibacterial effectiveness (Δlog_10_ CFU/mL) gave poor correlation (*R*^2^ = 68.02% for the C_max_/MIC and C_max_/MPC ratio, *R*^2^ = 39.42%, 57.62% for %T > MIC, %T > MPC, respectively).

### Magnitudes of PK/PD Parameters Determining Antimicrobial Effectiveness

**FIGURE 5:**
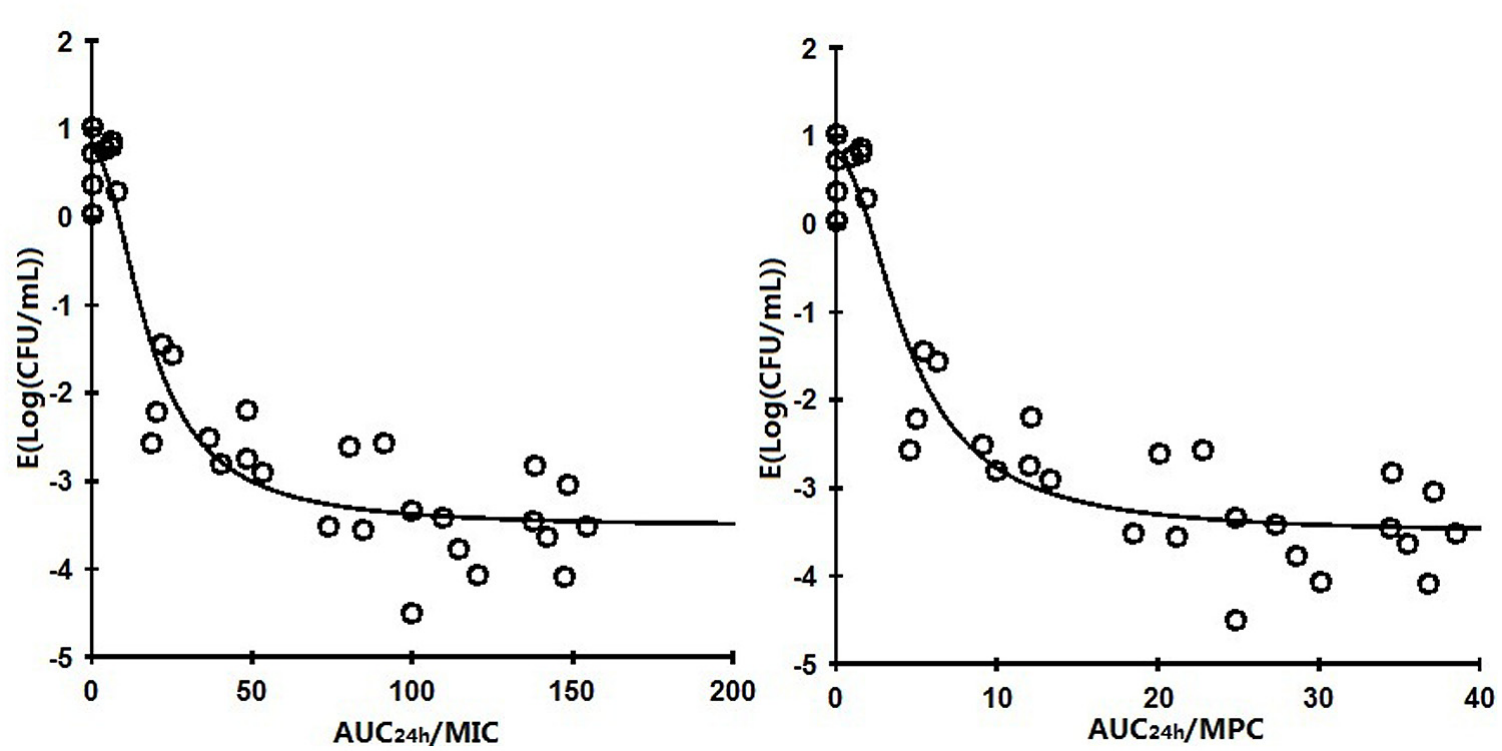
**Plots of AUC_24__**h**_/MIC or AUC_**24h**_/MPC ratio versus the difference of bacterial counts (Δlog_10_ CFU/mL) for *Pasteurella multocida* (CVCC1669) 24 h after i.m administration at various doses in the infected tissue-cage.** The line represents the curve of predicted values, based on the Sigmoid *E_max_* model, and the circles are the values for the individual tissue-cage.

The profile of Sigmoid *E_max_* model describing the relationship of antimicrobial effectiveness and the AUC_24h_/MIC or AUC_24h_/MPC parameter is presented in **Figure 5**. The values of AUC_24h_/MIC and AUC_24__h_/MPC for 1.5 log_10_ CFU/mL reduction (50% maximum response), 3 log_10_ CFU/mL reduction (90% maximum response) were 18.60, 50.65 h and 4.67, 12.89 h, respectively (**Table 2**). The slope of the AUC_24__h_/MIC ratio and AUC_24__h_/MPC ratio versus effectiveness was 1.92 and 1.85, respectively.

**Table 2:** *In vivo* PK/PD integration data obtained for marbofloxacin after i.m administrations at various doses in calves (*n* = 18).

PK/PD integration	Value
*E*_*max*_(Log_10_(CFU/ml))	0.74
*E*_*0*_(Log_10_(CFU/ml))	-3.57
*E*_*max*_-E_0_(Log_10_(CFU/ml))	4.28
AUC_24h_/MIC EC_50_(h)	18.60
AUC_24h_/MIC3log_10_CFU reduction(h)	50.65
AUC_24h_/MPC EC_50_(h)	4.67
AUC_24h_/MPC3log_10_CFU reduction(h)	12.89
Slope(*N*)	1.85

## Discussion

The understanding of PK/PD relationships of antimicrobials, and implications for rational dose setting, has increased considerably. However, most of the researches have been focused on human medicine. While it is probable that the general results are also applicable to the major animal species in veterinary medicine, the antimicrobial-bacterial combinations differ among animal species, due to difference in PK parameters (Zak and O’Reilly, 1991). So far, a variety of models have been expanding this area in veterinary medicine, such as *in vitro* dynamic model, *ex vivo* integrated model and the neutropenic murine model and so on(Andes and Craig, 2002a; MacGowan and Bowker, 2002; Mouton et al., 2005). Recently, tissue cage implanted subcutaneously models have been extensively developed to assist in identifying and defining the PK/PD parameter of antimicrobials (Greko et al., 2003a; Cui et al., 2006; Ni et al., 2014).

The PK/PD relationships of different fluoroquinolones against different bacteria have been extensively investigated both in *in vitro* dynamic model and in the different animal models. Most studies demonstrated that the AUC_24__h_/MIC ratio correlates significantly with the antimicrobial effectiveness while the C_max_/MIC ratio is associated with suppression of bacterial resistance (Drusano et al., 1993, 1998). In the present study, we found that AUC_24__h_/MIC is the PK/PD parameter predicting the antimicrobial effectiveness by non-linear regression analysis. The level of AUC_24__h_/MIC, that produced 50 and 90% of the maximum response corresponding to 1.5 log_10_ CFU/mL and 3 log_10_ CFU/mL reduction, were 18.60 and 50.65 h, respectively. The figures were lower than that for 90% maximal response reported in studies on marbofloxacin against *P. multocida* by *ex vivo* PK/PD integrated models while our studies adopted a higher inoculum (∼8 log_10_CFU/mL) compared with other investigation (∼6 log_10_ CFU/ml) at the start of experiments (Aliabadi and Lees, 2002; Sidhu et al., 2011; Potter et al., 2013; Shan et al., 2014). One of the explanations might be the importance of host defense mechanisms during treatment of infections caused by bacteria (Blaser et al., 1987). Likewise, the magnitude of the AUC_24__h_/MIC required for similar effectiveness is still lower than that observed in a neutropenic mouse pneumonia model (unpublished observations). The differences are caused by the shape of the marbofloxacin concentration profile *in vivo*, for the shape has an vital impact on antibacterial activity and the post antibiotic effect (PAE) *in vivo*, then contributes to the different magnitude of PK/PD parameter required similar effectiveness (Andes and Craig, 2002a; Rees et al., 2014). Besides, lower or higher figures have also been reported for different combinations of fluoroquinolones and bacterial species, both in *in vitro* dynamic models (Madaras-Kelly et al., 1996; MacGowan et al., 2000) and in murine thigh or lung infection models (Andes and Craig, 2002b), even in tissue cage models (Greko et al., 2003b), Jacobs (2001) showed that for concentration-dependent killing, the unbound serum AUC_24__h_/MIC ratio need to be >25–30 h for less severe infections or in immunocompromised patient. Thomas et al. (1998) found that an AUC_24__h_/MIC ratio of 125 h correlates with bacteriological cure for fluoroquinolones in human clinical trials and in laboratory animal infection models. For studies using tissue cages, Greko et al. (2003b) found that the values of AUC_24__h_/MIC producing 50 and 80% of the maximum response for danofloxacin against *Mannheimia haemolytica* are 101 and 244 h, respectively. Differences in study design, such as choice of measure of antimicrobial effectiveness, differences in inoculum at the start of experiments, PK parameters characteristic and different combination of antimicrobial and bacterial species, may explain the different values for antimicrobial efficacy reported in different studies (MacGowan and Bowker, 2002; Greko et al., 2003a). Besides, different bacterial isolates may vary the magnitude of AUC_24__h_/MIC required for antimicrobial effectiveness.

The most important risk factor for emergence of resistance is repeated exposure to suboptimal concentration of antibiotics (Zhao and Drlica, 2001). Some investigations indicated that the higher C_max_/MIC ratio (∼10 h or greater) is associated with a lower incidence of bacterial resistance (Drusano et al., 1993; Thorburn and Edwards, 2001). However, Drlica and Zhao (2007) proposed that the MPC is a possible application of the PK/PD approach for optimization of antimicrobial treatment regimens. Then, *in vitro* dynamic model and *in vivo* model studies (Olofsson et al., 2007; Liang et al., 2011; Ni et al., 2014) suggested that MPC-based PK/PD indices have an advantage over MIC-based indices for maximizing efficacy and minimizing selection of resistance. In our study, the value of AUC_24__h_/MPC ratio that is required near maximum effect was ∼13 h, while bacterial susceptibility is unchanged. In a previous study, *Escherichia coli* was treated with ciprofloxacin(Olofsson et al., 2007) and *Staphylococcus aureus* was treated with levofloxacin (Liang et al., 2011) for 24 h in an *in vitro* dynamic model, AUC_24__h_/MPC = 22 and 25 h correlated with restricted outgrowth of resistant mutant subpopulations, respectively. In the infected rabbit models, *in vivo* AUC_24__h_/MPC threshold corrected for protein binding of levofloxacin was 18 h for *Staphylococcus aureus* (Cui et al., 2006), and 14 h for *E. coli* (Ni et al., 2014), respectively. The differences were caused by different combination of antimicrobial and bacterial species. So far, because more and more studies indicated that the MPC-based PK/PD indices might be a more reliable PD parameter on evaluating antimicrobial dosing regimen, thus, our study made an attempt to provide the magnitude of AUC_24__h_/MPC required for different effectiveness for other researcher’s reference.

It is generally accepted that only the free fraction of antimicrobial in the interstitial fluid remains active against extracellular bacteria, but the bound fraction is generally recognized to be microbiologically inactive. Even if plasma and interstitial fluid levels of antibiotics do not always equilibrate, the concentration of unbound antibiotic in plasma approximates its free concentration in the extracellular space (*fu*_plasma_AUC_plasma_ = *fu*_interstitial_ _fluid_AUC_interstitial_ _fluid_, that is 48%AUC_plasma_ = 60%AUC_interstitial_ _fluid_; Drusano et al., 1998). Applying the value of AUC_plasma_ = 22.24 h•μg/mL following single intramuscular administration of 2 mg/kg body weight in diseased calves (Ismail and El-Kattan, 2007), we can calculate AUC_interstitial_ _fluid_ = 17.79 h•μg/mL at the infection site. Therefore, the PK/PD breakpoint based on MIC determined MHB can be calculated using following formula: AUC_interstitial_ _fluid_÷AUC_24__h_/MIC for a reduction of 3 log_10_CFU/mL ×*fu*_interstitial_ _fluid_ (Jacobs, 2001). Thus, we can conclude that the PK/PD breakpoint based on MIC determined MHB was 0.21 mg/kg, that is, the recommend dosing regimen of i.m administration at 2 mg/kg body weight per day will likely to be effective against *P. multocida* with marbofloxacin MICs of up to 0.21 mg/kg. A survey of marbofloxacin susceptibility of bacteria isolated from cattle with respiratory disease and mastitis in Europe in 2002–2008 indicated that MIC_90_ value of marbofloxacin against *P. multocida* is 0.12 mg/kg (Kroemer et al., 2012), we can infer that the recommended dosing regimen of marbofloxacin is likely very good effective against 90% or greater *P. multocida* isolates from bovine respiratory pathogen. Abo-El-Sooud and Goudah (2010) validated this point that the recommended dosing regimen of marbofloxacin has the favorable PD characteristics in diseased rabbits.

The PK/PD integration methods by tissue cage models display the advantage of allowing evaluation of antimicrobial activity and optimization of dosage regimens in the presence of the host defense, especially in the target animal of veterinary interest. In our study, we indirectly conclude that 2 mg/kg/day is likely to be effective against 90% or greater *P. multocida* isolates, according to AUC_24__h_/MIC values required for effectiveness at the infection site and MIC_90_ value of marbofloxacin against *P. multocida* (Kroemer et al., 2012). Of course, the results of this model need to be validated by clinical trials in relevant animal species; however, it is still a critical step between *in vitro* studies and clinical trials for the further understanding of PK/PD relationships of antimicrobials.

## Conflict of Interest Statement

The authors declare that the research was conducted in the absence of any commercial or financial relationships that could be construed as a potential conflict of interest.
